# Regulatory mechanism of circular RNAs in brain and neurodegenerative diseases

**DOI:** 10.3389/fnmol.2025.1507575

**Published:** 2025-05-16

**Authors:** Mandana Amelimojarad, Melika Amelimojarad

**Affiliations:** Department of Bioprocess Engineering, Institute of Industrial and Environmental Biotechnology, National Institute of Genetic Engineering and Biotechnology, Tehran, Iran

**Keywords:** Alzheimer's disease, biomarkers, circRNAs, non-coding RNA, neurological disease

## Abstract

Recent advancements in sequencing technology have allowed scientists to study the function and expression of different non-coding RNAs (ncRNAs). Circular RNAs (circRNAs) are increasingly recognized as prognostic and diagnostic biomarkers. These classes of ncRNAs are closed loops that are made from alternative mRNA splicing and can be found in large quantities with their evolutionarily conserved features across different species. These circRNAs can be derived from both coding and non-coding transcript and regulate cellular proteins and the expression of linear mRNA transcripts via their ability to act as miRNA sponges at various levels. Different circRNAs can affect the neurodegenerative disease pathogenesis such as Alzheimer's disease (AD), via altering a variety of methods. However, there is still limited knowledge about circRNAs in brain cells. Therefore, in this review, we will focus on the role of circRNAs in the brain to reveal a new potential therapeutic target and open the door to novel therapies and improved management of brain and neurodegenerative diseases.

## Introduction

Neurodegenerative diseases, which typically begin between the ages of 50 and 70, are slow-progressing conditions brought on by selective dysfunction and progressive damage to neurons in the central and peripheral nervous systems (Lamptey et al., 2022). The primary pathogenesis mechanisms of neurodegenerative diseases comprised of neuroinflammation, oxidative stress, aberrant autophagosome/lysosomal system, programmed cell death disruptions, persistent deposition of insoluble protein inclusion bodies in the cytoplasm or nucleus, including the formation of lewy bodies (LBs), β-amyloid (Aβ) deposition, and neurofibrillary degeneration (Afonso et al., 2008; Tapiola et al., 2009; Hinz and Geschwind, 2017; Sochocka et al., 2017). Circular RNAs (circRNAs) are a class of non-coding RNAs (ncRNAs) that are widely expressed in the cells, tissues, and organs of various species with their unique biological covalently closed structure and functions exhibiting cell or tissue specificity as well as developmental stage specificity (Chen and Shan, 2021; Zhang et al., 2024). The enrichment of circRNAs in the synapses of neurons can regulate the pathological process of neurodegenerative diseases via various signaling pathways since; they are generated more efficiently than their linear mRNA counterparts (Kristensen et al., 2017; Tang et al., 2024). Moreover, with the help of high-throughput total RNA sequencing, it was found that circRNAs have different regulation mechanisms than their linear counterparts (Chen and Schuman, 2016). CircRNAs have been found in a variety of body fluids, including blood, urine, extracellular vesicles, exosomes, and cerebrospinal fluid, according to earlier research (Wang and Fang, 2018). CircRNAs have been proposed as possible biomarkers for cancer and various other diseases because of their presence in these fluids (Fang et al., 2018; D'anca et al., 2022). The abundance of circRNAs and linear mRNAs that correspond to them is negatively correlated, according to several studies carried out on mammalian cells (Fang et al., 2018; Beilerli et al., 2022). This finding highlights the competition that arises during transcription because most circRNAs are transcribed from the same strand as their linear counterparts (D'anca et al., 2022). CircRNAs have shown a great deal of evolutionary conservation; their sequence and expression patterns are roughly 28% similar in mice and humans (Shen X. et al., 2022), with brain containing the majority of its concentration compared to other tissues, and the number of circRNAs steadily increases from embryonic to adult stages (Hansen et al., 2013; Shen X. et al., 2022). In the human brain, there are over 100,000 circRNAs expressed with an average of three circRNAs per gene (Memczak et al., 2013; Wang et al., 2014). These circRNAs are mainly produced from unique junctions within genes (Memczak et al., 2013; Dong et al., 2023). The presence of circRNAs in various biological processes and their involvement in human diseases, particularly neurological diseases, make them potential biomarkers and therapeutic targets. Therefore, by gaining a better understanding of their role in neurological pathways, highlighting their potential implications for understanding the diseases mechanisms and developing novel therapeutic strategies we can fill in the gaps and develop therapeutic approaches for future neurological diseases.

## Biogenesis of circRNAs

CircRNAs are distinguished from other non-coding RNAs based on their distinctive circular structure, their circular form is created by unique processes known as back-splicing, where a downstream splice donor is joined to an upstream splice acceptor, creating a closed-loop structure with covalent 3–5′ phosphodiester bond which is formed by joining a pre-mRNA'scanonical5′ and 3′ splice sites in reverse orientation with no free polyadenylated tail (Sharma et al., 2021; Xu et al., 2023). This unique arrangement not only protects them from degradation by exonucleases but also confers stability, allowing them to persist longer in the cellular environment than their linear counterparts (Sharma et al., 2021). The process is regulating by different trans-acting factors such as RNA binding proteins (RBPs), and Cis-acting elements (Memczak et al., 2013; Malviya and Bhuyan, 2023). Although the exact mechanisms are still unclear, it is believed that the spliceosome machinery directly contributes to the formation of circRNA, much like canonical splicing (Buerer et al., 2024). An internal back splicing event follows exon skipping in the intron lariat-driven circularization model, causing the lariat structure evades debranching which resulting in an mRNA devoid of the skipped exons. Primary elements that prevent the lariat from being broken down by debranching enzymes (such as Debranching RNA Lariats 1, DBR1), like a 7-nucleotide GU motif and an 11-nucleotide C-rich sequence close to the 5′ splice sites, encourage the formation of circRNA (Martin et al., 2013; Pisignano et al., 2023). By modifying RNA through adenosine to inosine (A-to-I) conversions, adenosine deaminases acting on RNA (ADARs) contribute significantly to circRNA biogenesis. The base-pairing of reverse complementary sequences, which alters the stability of RNA secondary structures, serves as the basis for this editing (Erdmann et al., 2021). ADARs control the accessibility of regulatory RBPs by changing these structures, which either decreases or increases back-splicing events (Shen H. et al., 2022).

Furthermore, circRNA biogenesis has been linked to N6-methyladenosine (m6A) RNA modification, which is known to control a number of RNA metabolism processes, such as translation, stability, and splicing. In particular, the nuclear m6A reader protein YTHDC1 (YTH N6-Methyladenosine RNA Binding Protein C1) can back splice m6A-modified exons that are close to the start and stop codons of mRNAs (Tang et al., 2020; Dattilo et al., 2023).

## CircRNAs characteristics and localization

The emergence of circRNAs is one of the more exciting developments in molecular biology, offering new insights into gene regulation and cellular processes (Mo et al., 2021). By fine-tuning circRNA levels, cells can adapt to changing conditions, ensuring that appropriate proteins are synthesized in response to signaling cues. Therefore, understanding both stability and degradation pathways is essential for deciphering the regulatory networks in which circRNAs operate (Salzman, 2016). The diversity in circRNA expression patterns can significantly influence cellular functions and pathways, making their stability a critical aspect of gene regulation. More precisely, circRNAs can be derived from exons (ecRNA) or introns (ciRNAs), the predominant class, or both exon-intron (ElciRNAs) (Li X. et al., 2018). The nucleus-based circRNAs also can control transcription and splicing as well as the transcription and post-transcriptional levels of gene expression. CircRNAs can also attach to various proteins to form distinct circRNA-protein complexes (circRNPs), which in turn control the activity of the associated protein, the subcellular location of proteins, and the transcription of related or parental genes. However, additional research is required to fully investigate the world of circRNA (Li X. et al., 2018; Feng et al., 2023). Exonic-derived circRNAs make up the majority of circRNAs, prefer to be found in the cytosol, and it seems that their length can influence nuclear export (Kristensen et al., 2019), intronic and some exonic circRNAs also preferentially concentrate and function in the nucleus (Li et al., 2015; Kristensen et al., 2019).

One of the remarkable features of circRNAs is their heightened stability compared to linear RNA counterparts. The closed-loop structure of circRNAs protects them from exonuclease activity, which typically leads to degradation of linear RNAs. This enhanced stability allows circRNAs to persist within cells for extended periods, thereby increasing their potential to impact gene expression regulation. Several studies have demonstrated that many circRNAs are expressed at higher levels than their linear counterparts, suggesting a functional advantage conferred by their stable nature (D'Ambra et al., 2019; He et al., 2021; Sharma et al., 2021). Since most known circRNAs are produced by RNA polymerase II transcription of protein-coding genes, their stability is predicted to be 2.5–5 times higher than that of linear transcripts (You et al., 2015; Li X. et al., 2018). The stability of circRNAs is not uniform across different types and cellular conditions. Various studies have revealed that factors such as the length, sequence composition, and the cellular environment can influence circRNA stability. Shorter circRNAs tend to be less stable, while those with specific nucleotide sequences or structural motifs may enjoy enhanced stability. Furthermore, environmental stressors, such as oxidative stress or hypoxia, can impact the turnover rates of circRNAs, potentially altering their expression profiles and functional roles (Buerer et al., 2024). Beside stability, degradation processes of circRNAs are also crucial for elucidating their functional roles in various biological contexts. This resilience is attributed to their covalently closed structure, making it challenging for ribonucleases, which rely on these features for recognition and cleavage, to degrade circRNAs efficiently (Feng et al., 2023). One of the primary mechanisms for circRNA degradation involves endonucleolytic cleavage, which can occur when specific ribonucleases recognize and bind to certain circRNA motifs (39). The exonucleases RNA interference (RNAi) pathways that specifically degrade circRNAs has been linked to the Argonaute-2 (AGO2) endonuclease, which is guided by different miRNAs to inhibit inhibits circ RNAs expression in an AGO2-dependent way by binding to the precursor circRNAs in the nucleus of mouse spinal cord neurons. This targeted degradation allows the cell to modulate circRNA levels in response to various stimuli, enabling fine-tuning of their regulatory activities (Pan et al., 2019).

Interestingly, the circRNAs can be degraded with RNA-binding proteins (RBPs) for example, certain RBPs like UPF1 may bind to specific regions of circRNAs, unwinding their structure and helping the enzymatic degradation within cells (Li et al., 2015; Fischer et al., 2020).

Another layer of regulation is introduced by microRNAs (miRNAs). miRNAs can interact with circRNAs, helping their degradation. Conversely, some circRNAs exhibit sponge-like behavior, sequestering specific miRNAs, thus preventing them from exerting their effects on target mRNAs. This interaction further highlights the complex landscape of circRNA stability and degradation, showcasing their dual role in gene regulation (Pan et al., 2019).

## The mechanisms of action of circRNAs

CircRNAs have emerged as significant regulators of gene expression, showcasing a diverse array of functions that challenge our traditional understanding of RNA biology (Li X. et al., 2018), although traditionally, it was thought that only messenger RNAs (mRNAs) could be translated into proteins (Huang et al., 2020). The prominent functions of circRNAs include the regulation of genes expression, protein translation, and transcriptional regulation (Sharma et al., 2021), which play important and conservative roles in living organisms (Ma et al., 2023).

CircRNAs can contain multiple binding sites for specific miRNAs, effectively sequestering them away from their native mRNA targets, this sponge-like activity results in a decrease in the availability of miRNAs, thus alleviating their repressive effects on target mRNAs (Garikipati and Uchida, 2021). The interplay between circRNAs and miRNAs adds a complex layer of post-transcriptional regulation, influencing various biological processes such as cell proliferation, apoptosis, and differentiation (Zhu et al., 2022).

For example, the circRNA ciRS-7 has been shown to bind and inhibit miR-7, a miRNA implicated in several neurodegenerative diseases and cancers (Shi et al., 2017). By sponging miR-7, ciRS-7 enhances the stability and expression of miR-7's target genes, thereby affecting cellular signaling pathways and promoting tumor progression (Shi et al., 2017; Shao et al., 2023). This mechanism underscores how circRNAs contribute to the fine-tuning of gene expression levels and can have profound implications on health and disease. In addition to these primary mechanisms, circRNAs can also interact with proteins, forming ribonucleoprotein complexes that influence various pathways in the cell (Huang et al., 2020).

Recent studies have demonstrated that certain circRNAs can also serve as templates for translation, particularly in specific cellular contexts (Su et al., 2024). For instance, circRNAs can possess internal ribosome entry sites (IRESs) or other elements that facilitate the recruitment of the ribosomal machinery, allowing them to be translated into peptides or proteins. This was first highlighted with the discovery of circRNA derived from the CDR1 gene, which encodes a functional protein believed to play a role in neuronal function (Shao et al., 2023).

**Figure 1:**
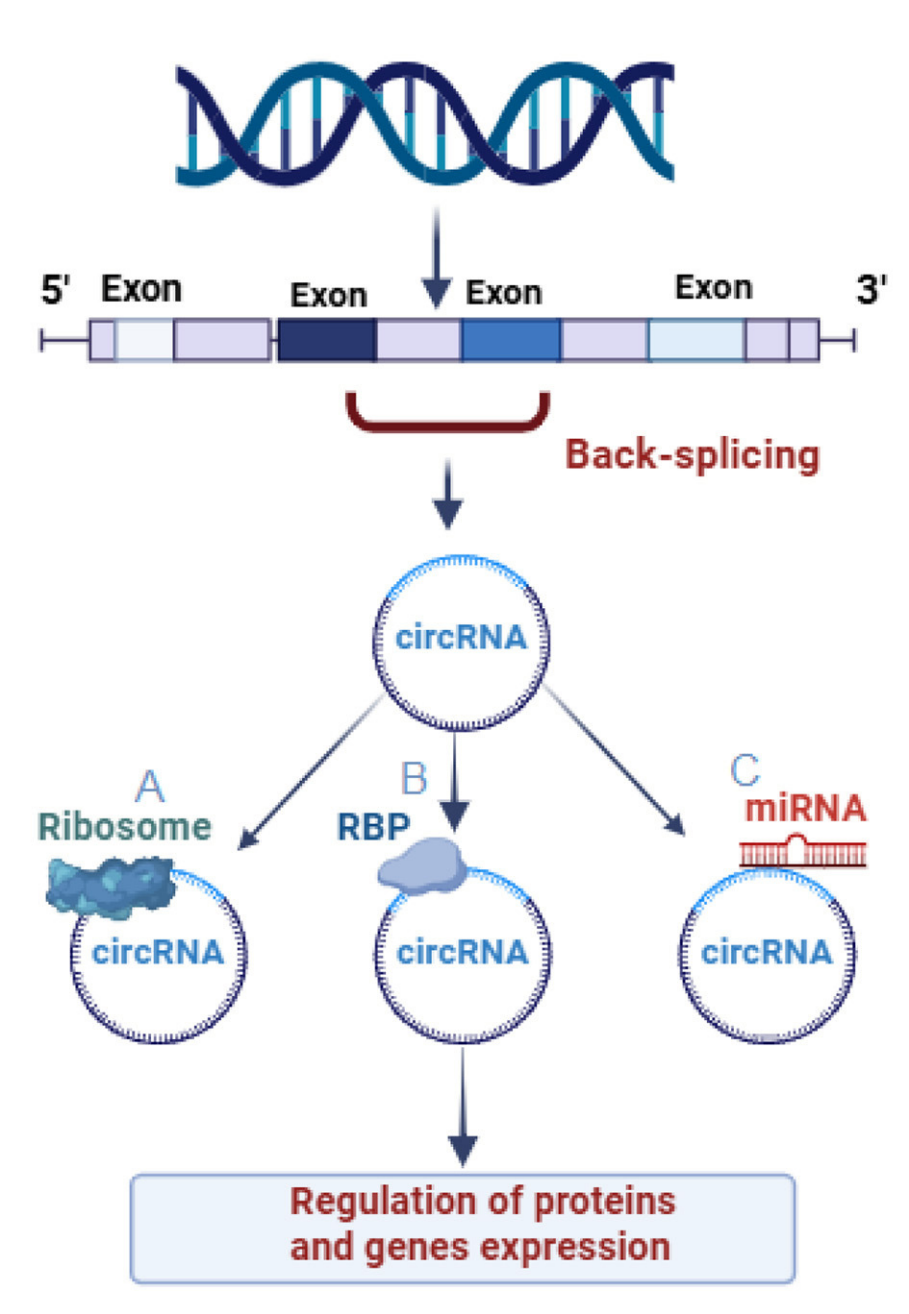
Function of three differently generated Circular RNAs (circRNAs). **(A)** CircRNAs which participate in protein translation, modifying the transcription, and protein-coding function**. (B)** CircRNAs attached to RNA binding proteins. **(C)** CircRNAs participate in controlling the activity of miRNA-by-miRNA sponge's function.

According to a number of studies, a subset of circRNAs may be essential for delivering RBPs to particular subcellular sites, assisting in the formation of complexes between proteins and RNA and enzymes, and functioning as protein sponges or decoys, for instance circYAP1 overexpression is associated with decreased immune activation against cancer cells in colorectal cancer. Immune evasion and tumor progression result from CircYAP1's direct binding to the YAP1 (yes1 associated transcriptional regulator) protein, which inhibits its phosphorylation and increases YAP1 nuclear import. Interactions with transcription factor 4 (TCF4) then stimulate the expression of the immune checkpoint inhibitor PD-L1 (CD274) (Huang et al., 2020; Chen et al., 2023). CircRNAs have a number of ways to control the transcription of genes, certain circRNAs function as either positive or negative transcriptional regulators by directly interacting with elements of the RNA Polymerase II (Pol II) complex (Huang et al., 2020), for example, a recent study showed that circRNAs carrying metal-sensitive elements prevent gawky, a chromatin-interacting RBP, from being recruited to active chromatin regions, hence inhibiting the transcription of genes responsive to copper stress, this obstruction causes an abnormal buildup of gawky in the cytoplasm, which interferes with the transcription of genes (Cao et al., 2024). Or they can help the proteins degradation by binding to specific RBPs, for example, circDNMT1 can help the nuclear translocation of the transcription factor TP53 (tumor protein P53), and RBP AUF1 (HNRNPD, heterogeneous nuclear ribonucleoprotein D) proteins in breast cancer (Du et al., 2018) (Figure 1).

## CircRNAs identification with computers

Recent advancements in bioinformatics algorithms have enhanced our ability to identify, validate circRNAs, and providing valuable insights into their biological roles. These technologies provided us with an increased probability of uncovering new circRNAs by rescuing numerous unmapped reads through chimeric mapping in a back-splicing order (Xia et al., 2017; Ragan et al., 2019). These technological advancements have successfully identified robust associations between the mis regulation of circRNAs and various diseases (Memczak et al., 2013; Xia et al., 2017; Ragan et al., 2019).

Recently, with the help of the new bioinformatic tools for circRNAs analysis, we can provide comprehensive information about their origin including chromosome coordinates, cell types, and also predict splicing sites, miRNA binding sites or RNA-binding proteins sites. considering that there is no single “gold standard” tool for circRNA identification, and most computational tools for circRNA identification rely on initial experimental evidence from RNA-seq data to enhances accuracy of the results for finding circRNAs, combining multiple detection methods is needed (Huang et al., 2020). At the moment computational approaches to circRNA identification are divided into two main strategies: pseudo-reference-based and chimeric-read-based, pseudo-reference-based methods to create back splice junctions' references, use existing gene annotation data, notable pseudo-reference-based tools include: NCLscan, KNIFE, and Circall which applies a two-step pseudo-reference approach to reduce false positives results, it first maps RNA-seq reads to an annotated transcriptome reference to remove read of linear RNA, then aligns the unmapped reads to back splice junctions' references database (Nguyen et al., 2021). While in Chimeric-read-based methods such as CIRI2, DCC, CIRCexplorer3, and circRNA finder, directly align each sequence reads to a reference genome, using non-colinear or chimeric reads in order to find back splice junctions. miRanda, TargetScan, and RNAhybrid consider as the databases repurposed for prediction of circRNA-miRNA interaction. CircInteractome, circRNADb, C2CDB, CircSSNN, CRAFT, cirCodAn, CircSite, circAtlas, and catRAPID omics v2.0 are used for circRNA-RBP interaction prediction. CIRI-vis and circView are visualization tools support the analysis of splicing patterns, miRNA/RBP binding sites helping us comprehend the structure and functionality of circRNAs. For circRNA identification, RNA sequencing is still the most common technique; however, precision and specificity are limited by a lack of standardized protocols and detection tools. Research communication and replication are made more difficult by the lack of a standardized nomenclature for circRNAs. Although conversion tools have been added to databases like circBank to address this problem, incomplete reporting of circRNA transcript lengths—often as a result of short-read sequencing limitations—remains a major obstacle. These restrictions are starting to be addressed by long-read sequencing technologies in conjunction with sophisticated bioinformatic tools such as CIRI-long, which allow for the accurate identification of circRNA structures, interaction sites, and functional roles (Memczak et al., 2013; Xia et al., 2017; Ragan et al., 2019; Huang et al., 2020; Rebolledo et al., 2023).

## CircRNAs in the brain and neurological conditions

Research has demonstrated that circRNAs are present in large quantities and exhibit high expression levels in the central nervous system, particularly in nerve synapses (Rybak-Wolf et al., 2014). This is observed during the process of cellular differentiation as well as in response to bursts of electrical activity (Rybak-Wolf et al., 2014). Approximately 60% of circRNAs in the central nervous system are upregulated during development, with a significant emphasis on synaptogenesis (Rybak-Wolf et al., 2014). In contrast, only 2% of their linear isoforms display a similar trend, suggesting that circRNAs may play a role in regulating synaptic plasticity, neuronal development, aging, and human neuropsychiatric diseases (Rybak-Wolf et al., 2014; Gokool et al., 2020). Brain cells have the remarkable ability to generate numerous circRNAs, particularly in regions where linear mRNA expression lacks diversity (Gokool et al., 2020). This phenomenon leads to the creation of a diverse transcriptome and a wide range of biological pathways, ultimately contributing to the performance of human brain cells (Dube et al., 2019; Gokool et al., 2020). While circRNAs are predominantly found in the cytoplasm, a significant portion of them is localized to and enriched in synapses (Rybak-Wolf et al., 2014; Dong et al., 2023). Furthermore, it has been observed that the levels of circRNAs are higher in the human fetal brain compared to other fetal tissue types or even in the adult human brain. This finding suggests the potential significance of circRNAs in brain development (Hansen et al., 2013; Dong et al., 2023). CircRNAs exhibit varying levels of expression across different types of brain cells (Rybak-Wolf et al., 2014).

Notably, an increase in circRNA expression has been observed during the process of neuronal differentiation, particularly in forebrain neuron progenitor cells and the frontal cortex (D'Ambra et al., 2019). It is worth mentioning that the expression of circRNAs, when compared to their corresponding linear transcripts, tends to be lower and more cell-specific (D'Ambra et al., 2019; Ragan et al., 2019).

CircRNAs are expressed in human brain tissue and have age and gender-specific patterns of expression (Jakobi and Dieterich, 2018). Recent research has revealed a fascinating connection between diet, and ncRNAs in the field of cancer therapy (Jakobi and Dieterich, 2018). Notably, studies have shed light on this important link and, it has been observed that both diet and stress can have a profound impact on the expression profiles of circRNAs in the brain cortex (Chen and Schuman, 2016).

For instance, a high-fat diet-induced diabetes has been found to alter the levels of circRNAs in adult mice (Memczak et al., 2013; Jakobi and Dieterich, 2018; Das and Ganesh, 2023). Additionally, significant alterations in the expression of circDYM have been observed in the blood of patients with chronic unpredictable stress and MDD (Yu et al., 2022). These findings highlight the intricate relationship between diet, stress, and the expression of circRNAs, providing valuable insights for potential therapeutic interventions (Yu et al., 2022). Up to 36 neuronal circRNAs are produced by various genes, with a large proportion originating from the synapse machinery and synaptic genes, according to a recent study on circRNA in the brain (Dong et al., 2023). This study is further supported by findings indicating that the level of circRNA is reduced in cultures comprising a substantial population of glial cells. This suggests that the observed increase in circRNA expression may be exclusive to neurons or potentially linked to interactions between neurons (Dong et al., 2023).

CircRNA expression in subcellular compartments like the cell body and dendrites, micro-dissected neuropil within hippocampus slices, and synaptosomes suggests that circRNAs may be involved in the translation of mRNA within neurons and have an impact on mRNA levels. This suggests that, especially through synaptic or dendritic content, circRNAs are essential for controlling the correct transport and translation of mRNAs in neurons. As a result, they greatly influence neural activity and are necessary for adaptive reactions to diverse stimuli (Venø et al., 2015; Gokool et al., 2020). It's interesting to note that synaptic circRNAs are implicated in most brain disorders. These circRNAs influence synaptic dysfunction or synaptopathy by changing the translation and localization of mRNA (Gokool et al., 2020). This early abnormality in many brain tumors and neurodegenerative diseases emphasizes the importance of circRNAs in these disorders (Chen and Schuman, 2016).

CircRNAs have been identified as crucial mediators in the progression of glioma, influencing various cellular processes such as proliferation, invasion, migration, and apoptosis. Several circRNAs, including circMTO1, circHIPK3, and circMMP9, have been found to promote glioma progression by sequestering miRNAs and targeting their host genes (Wang et al., 2018; Zhang X. et al., 2019; Zhou et al., 2021). This knowledge highlights the significance of circRNAs in the development and advancement of glioma. The circSMARCA5 molecule plays a role in promoting angiogenesis and the progression of glioma by regulating the mRNA of VEGF-A, which is known to be a powerful inducer of angiogenesis. It achieves this by interacting with the serine and arginine-rich splicing factor 1 (SRSF1) (Barbagallo et al., 2019).

On the other hand, circZNF292 down-regulates various components of the Wnt/β-catenin signaling pathway and induces hypoxia (Yang et al., 2016). Another circular RNA, circ_002136 (also known as circCDK11A_001), appears to regulate the expression of SOX13, which in turn induces the expression of the spondin-2 gene (He Z. et al., 2019). This gene is associated with tumor aggressiveness and contributes to the promotion of glioma angiogenesis (He Z. et al., 2019).

CircTTBK2 by targeting HNF1β/Derlin-1 pathway prevented cell apoptosis and thereby, promoted proliferation, migration, and invasion of glioma cells (Zheng et al., 2017). The circ_0046701 increases integrin subunit beta 8 (ITGB8) by sequestering miR-143–3p and thereby promoting glioma invasion (Li G. et al., 2018). We have summarized the top list of top circRNAs in brain pathology in Table 1.

**Table 1:** Role of top studied circRNAs in brain pathobiology.

**Gene**	**Function**	**Samples**	**References**
circ-001372	Decreases neural apoptosis and inflammation through PIK3CA/Akt/NF-kappaB via miRNA-148b-3p	Sprague Dawley rats	Lin and Wan, 2022
circTTBK2	Known to promote angiogenesis and glioma progression	glioma	Zheng et al., 2017
circMMP9	Known to promote angiogenesis and glioma progression	glioma	Wang et al., 2018
circ-0000220	Increase production of inflammatory cytokines	BV-2 microglial cells	Zhang Y. et al., 2019
circRNA circZNF292 (cZNF292)	Induced by hypoxia down-regulates various components of the Wnt/β-catenin signaling pathway,	glioma cells	Yang et al., 2016
circ_002136	Regulate angiogenesis in glioma miR-138-5p/SOX13	glioma cells	He Z. et al., 2019
circ-KIAA1586	Regulate pathological process of AD via miR-29b, miR-101, miR-15a	glioma cells	Zhang Y. et al., 2019
circRNA_0000950	Sponge miR-103 sponge and regulate neuro-inflammation and apoptosis	PC12 cells	Yang et al., 2019

## CircRNAs in Parkinson's disease

Parkinson's disease (PD) is the second most prevalent neurodegenerative disorder after AD which can affect around 1–2% of individuals worldwide (Cherian and Divya, 2020). This neurodegenerative disorder primarily affects movement and can lead to a range of debilitating symptoms including tremors, rigidity, and bradykinesia, while the exact cause of Parkinson's disease remains elusive, research continues to unveil potential genetic and environmental factors contributing to its onset (Cherian and Divya, 2020). PD is clinically recognized as motor deficit cognitive decline causing movement problems by inducing the death of more than 50% of dopaminergic neurons inside the substantia nigra (SN) (Hanan et al., 2020), leading to motor and non-motor symptoms. Although the exact reasons behind the degeneration of dopaminergic neurons in PD are not well comprehended. The underlying causes of PD are complex, involving genetic, environmental, and lifestyle factors. Recent advances in genomic technologies have unveiled a diverse array of non-coding RNAs, among which circRNAs appear to play crucial roles in gene regulation and cellular function. Unlike linear RNAs, circRNAs form closed-loop structures that confer stability and can interact with various proteins and RNA molecules (Ravanidis et al., 2021).

CircRNAs, has emerged as a significant player in the field of molecular biology, particularly in the context of various neurological disorders, including PD, and may hold significant implications for understanding and potentially treating therapy (Hanan et al., 2020).

It has been proved that 5–10% of PD are familial and can be influenced by a pattern of genetic inheritance such as autosomal-dominant in (LRKK2, SNCA, VPS35) and autosomal recessive mutation in (DJ-1, PINK1, Parkin) (Cherian and Divya, 2020; Kim and Jeong, 2020). Besides genetic mutation, male gender, aging, exposure to environmental toxins, excessive oxidative stress, mitochondrial malfunctioning, imbalanced signaling pathways, and cell death can affect sporadic initiation of PD and induction of LBs (Kim and Jeong, 2020).

LBs are the cytoplasmic fibrillar inclusions in the SN region considered the main histological hallmark of PD since neuronal loss has been increased at the LBs' predilection cited, however, it's important to note that their formation is not the main cause of cell death (Wakabayashi et al., 2007; Tysnes and Storstein, 2017).

So far, over 70 molecules have been found in LBs formation but still, α-synuclein (α-syn) is considered a major component of LBs and it is now established that α-synuclein plays a significant role in LBs in both hereditary and sporadic PD, and its higher seeding kinetics can exhibit a unique role in perturbations of proteomics profiles of mitochondria and lipid metabolism (Kon et al., 2024). After the discovery of various non-coding RNAs (ncRNAs) and their biological implications on several neurodegenerative disorders as an important regulator, aberrant RNA metabolism specially circRNAs based on their structure, stable expression, and their essential role in controlling gene expression especially modulating α-syn expression as the abundant neuronal protein connected to the etiology of many neurodegenerative diseases, and also the fact that a high number of brain-enriched circRNAs have been linked to the pathogenetic mechanisms underlying neurodegeneration and aging-related diseases, gaining further insight into the circRNA-α-syn regulatory mechanism may offer a possible therapeutic target for PD treatment. Here we discuss the latest work on identifying the circRNAs in PD in brief (Kon et al., 2024).

In a recent study, scientists by using the most recent PD Genome-Wide Association Studies (GWAS), looked up the genes that at least produce one or more circRNAs and indicated that CircDNAJC6 which exhibited high expression levels in dopamine neurons, and moderate levels in pyramidal neurons was reduced at the earliest prodromal PD (Kon et al., 2024).

The discovery of mutations connected to parkinsonism has brought attention to the DNAJC protein family, a subclass of heat shock proteins, the process of uncoating clathrin-coated synaptic vesicles is significantly aided by DNAJC649 therefore the lower level of this gene and its generated circRNAs can balance neuronal integrity and neuronal survival (Roosen et al., 2019).

Additionally, circRNAs can regulate the expression of genes involved in neuroinflammation and apoptosis, they can significantly contributing to the pathological processes observed in PD and influence neuronal health and the progression of the disease (Wang M. et al., 2021; Xiao et al., 2024). Moreover, changes in the expression levels of specific circRNAs have been correlated with the dysregulation of critical genes involved in dopamine metabolism and mitochondrial function. These alterations can exacerbate the neurodegenerative processes associated with Parkinson's disease, paving the way for further research into their therapeutic potential.

CDR1, a circRNA highly expressed in excitatory neurons, can regulate miR-7. It has been observed that PD patients have lower levels of miR-7 in the SN region, which is associated with a higher accumulation of α-Syn in LBs, since the binding of miR-7 to the 3′UTR of α-syn directly inhibits its expression, indicating that miR-7 downregulation may play a role in α-syn aggregation, therefore upregulation of other circRNAs targeting miR-7 such as cirs-7 or circ-SNCA hosted by the SNCA gene which encodes α-syn protein can play a regulating role in the enrichment pattern of nucleoprotein in role in PD (Liu et al., 2022).

Following pramipexole (PPX) treatment, a dopamine D2/D3 receptor agonist, the expression levels of SNCA and circSNCA in PD were downregulated. The downregulation of the SNCA gene caused by circ-SNCA knockdown was linked to a decrease in apoptosis and an increase in autophagy via miR-7 sponge. Therefore, circ-SNCA inhibition could delay the worsening of the disease and serve as a potential therapeutic target of PD (Sang et al., 2018). CircZip-2, derived from the Zip-2 gene, is another circRNA that indirectly regulates α-Syn levels and its expression decreases in PD (Ghosal et al., 2013).

Higher expression of circRNA derived from the pantothenate kinase 1 (Pank1) gene (circ-Pank1) was also detected in the SN of PD model mice treated with rotenone, which was also related to the damage to dopaminergic neurons *in vivo* and *in vitro*. circ-Pank1 by sequestering miR-7a-5p upregulate the expression of α-synuclein (α-syn), so far, circRNA profiling in both PD and healthy controls has identified a large number of differentially expressed (DE-circRNAs) that accumulate with aging in the brain and are associated with PD pathology by influencing the stability and transport of miRNAs, impacting the expression of α-synuclein (α-Syn), oxidative stress, aberrant mRNA transcription, cell death, and autophagy. These DE-circRNAs have been demonstrated to regulate every facet of neuronal and glial function, and there is evidence to suggest that they could be used as prognostic and diagnostic biomarkers according to risk score formulas and ROC curve analysis (Jia et al., 2020; Liu et al., 2022).

The microarray-based analysis of the plasmatic circRNAs of three PD patients compared to the controls revealed that the circ_0004381 and the circ_0017204 (referred to as circARID1B and circTCONS-l2-00002816) could predict PD at an early diagnosis with relatively high sensitivity and specificity, while circ_0085869, circ_0004381, and circ_0090668 (also known as circHUWE1, circFAM83H, circARID1B) might be able to distinguish between late-stage and early-stage of PD (Zhong et al., 2021).

According to Hanan et al., different brain regions expressed different combinations of 24 circRNAs. Of them, the increase in circSLC8A1 expression is most strongly linked to the pathological modifications of Parkinson's disease (PD) caused by excessive oxidative stress, which affects Nrf2, a critical antioxidant transcription factor, and may be one of the causes of dopaminergic neuron damage (Anandhan et al., 2021).

The SLC8 gene family encoding, Na+-Ca2+ exchanger (NCX) isoforms constitute the major cellular Ca2+ extruding system in neurons and microglia. Exposure of SH-SY neuronal cells with the Paraquat (PQ) oxidative reagent, induced higher circSLC8A1 in a dose-dependent manner while the decreased Solute Carrier Family 8 Member A1 (SLC8A1) protein levels, also circSLC8A1 via regulating miR-128 is linked to PD modulating oxidative stress (Hanan et al., 2020).

The over expression of circFTO (Hsa_Circ_0105596) with ability to target and inhibit miR-187-3p in SH-SY5Y cells causes EEF2 altered expression in PD, which can promote the advancement of PD, while circFTO interference can successfully decrease brain inflammation and lower oxidative stress (Feng et al., 2024).

**Figure 2:**
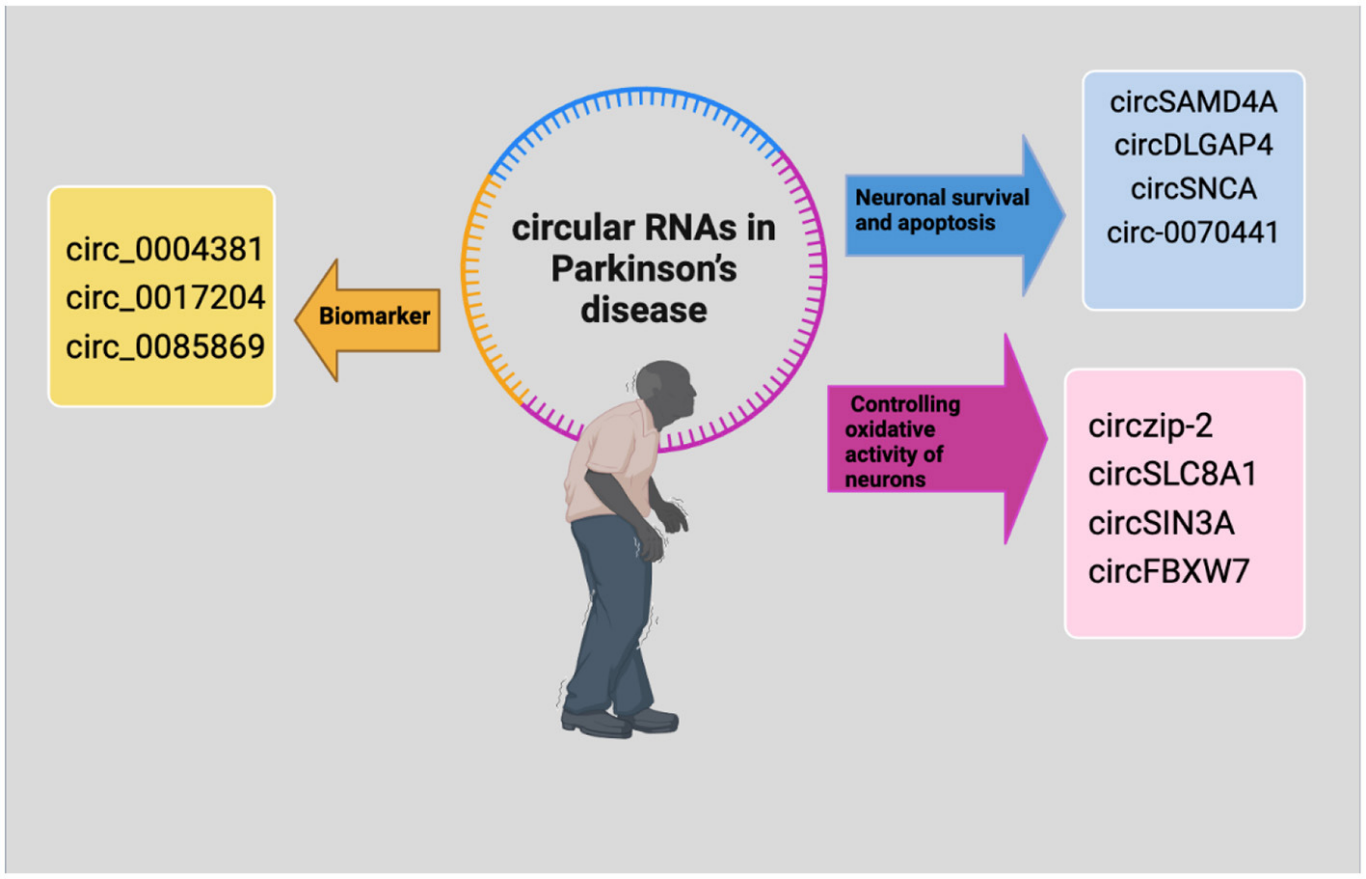
The regulatory mechanisms of circRNAs in Parkinson's disease. CircRNAs that control the oxidative activity of neurons are indicated by purple, circRNAs with biomarker potential are indicated by yellow and those that are controlling neuronal survival and apoptosis are indicated by blue.

In conclusion, circRNAs represent a novel and exciting frontier in the research landscape of PD. Their involvement in gene regulation, cell survival, and potential as biomarkers underscores their importance in the disease's pathophysiology. As research continues to unravel the complexities of circRNA functions, they may hold the key to understanding and ultimately treating PD, offering hope for improved management and outcomes for affected individuals. The top circRNAs interaction in PD development is demonstrated in Figure 2 and Table 2.

**Table 2:** Role of top studied circRNAs in the PD pathogenesis.

**CircRNAs in AD**	**Target**	**Function**	**References**
circDLGAP4	miR-134-5p	Neuroprotection	Bai et al., 2018
circSAMD4A	miR-29c-3p AMPK/mTOR	Apoptosis and autophagy	Wang W. et al., 2021
circSNCA	miR-7	Apoptosis and autophagy	Sang et al., 2018
circSLC8A1	miR-128 miR-132 Sirt-1	Neuroinflammation	Hanan et al., 2020
mmu_circ_000329, mmu_circ_000132, mmu_circ_000597, mmu_circ_000538, mmu_circ_0003292	miR-132		Yang et al., 2012
Circzip-2	miR-60	Diagnostic effect	Kumar et al., 2018
CircTLK1	miR-26a-5p/DAPK1	Improving the neurological dysfunction	Chen et al., 2022
Circ_0070441	miR-626	Reducing the cellular neurotoxins	Cao et al., 2022
Circ-SIN3A		Response to oxidative stress	Kong et al., 2021
Circ-FBXW7		Response to oxidative stress	Kong et al., 2021
Hsa_Circ_0105596/FTO	miR-187-3p EEF2	PD progression	Feng et al., 2024

## CircRNAs in Alzheimer's disease

Alzheimer's disease is a progressive neurodegenerative disorder that primarily affects memory, thinking, and behavior (Levites et al., 2023). It is the most common form of dementia, accounting for 60%−80% of cases. As the population ages, the prevalence of Alzheimer's disease continues to rise, becoming a significant public health concern (Levites et al., 2023). While the exact causes of Alzheimer's are still not fully understood, research into genetic, environmental, and cellular factors has illuminated some pathways that might contribute to the onset and progression of this disease (Husain et al., 2021). The majority of research on neurodegenerative diseases has concentrated on the roles of circRNAs in AD (Mo et al., 2020), based on its impact on global public health. In general, patients with AD experience intellectual disabilities and memory loss (Mo et al., 2020). The development of Aβ-42 plaques, neurofibrillary tangles (NFTs) made of hyperphosphorylated tau protein, and neuroinflammation brought on by the activation of microglia cells are the main pathological features of AD (Congdon and Sigurdsson, 2018; Bălaşa et al., 2020). Nemour studies showed that circRNAs directly contribute to the pathology and progression of AD by controlling the brain's inflammatory responses, tau protein accumulation, and Aβ metabolism (Fang et al., 2018; Dolinar et al., 2019).

Multiple expressed circRNAs were detected in AD models, playing a role in signaling pathways and targeting genes by miRNA sponges, therefore studying circRNAs is a key approach to understanding their important epigenetic regulatory mechanisms in the central nervous system pathogenic gene expression program (Tang et al., 2024). Currently, the most researched circRNA, CDR1as has been linked to several cancer pathologies as well as AD.CDR1 exhibited a potent miRNA sponge function, to regulate downstream target gene levels. For example, CDR1 was found to be downregulated in AD sporadic patients, which may upregulate the expression of miR-7 and lead to ubiquitin-conjugating enzyme E2A (UBE2A) depletion which normally coordinated the removal and degradation of damaged and amyloid-containing proteins by 26S proteasomes (Fagan et al., 2007; Zhao et al., 2016; Shao et al., 2023). Interestingly, the neuroprotective role of the same circRNA was studied by promoting amyloid Precursor Protein (APP) and Beta Secretase 1 (BACE1) protein degradation via the proteasome and lysosome pathways (Sakae et al., 2016; Shi et al., 2017). Likewise, circHDAC9 has been found to have a neuroprotective function by sponging mir-142-5p, the upregulation of this circRNA reduced the neuronal damage caused by Aβ (42) in human neuronal (HN) cells (Shen X. et al., 2022). It's important to note that berberine treatment, could increase circHDAC9 expression and protect HN cells from Aβ-42 neuronal damage (Lu et al., 2019).

**Figure 3:**
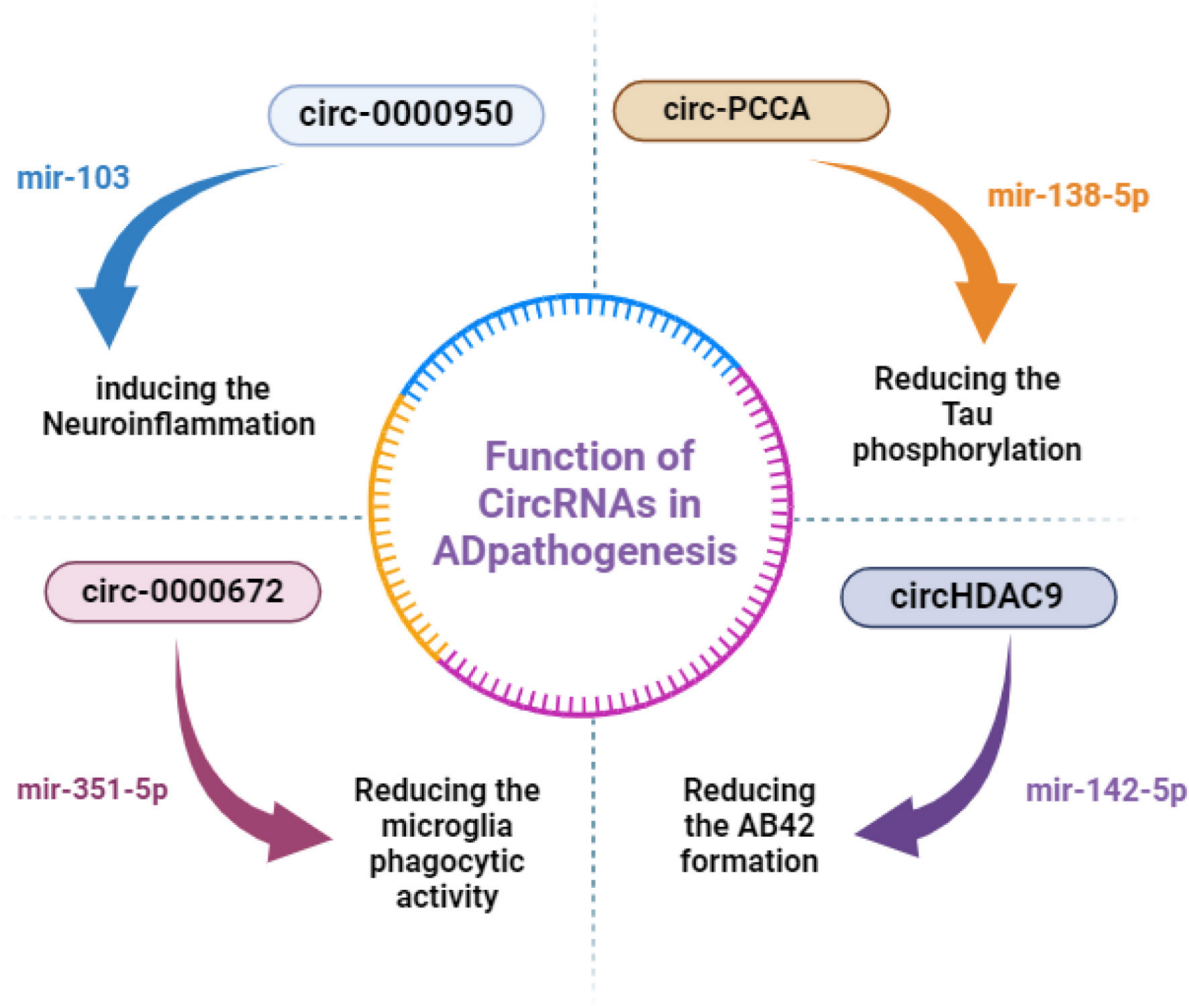
CircRNAs' regulatory mechanisms in AD. Non-coding RNAs that are upregulated are indicated by red words, and those that are downregulated are indicated by blue words. miR, microRNA; circRNA, circular RNA.

CircHOMER1, which is produced by the HOMER1 gene, is highly intriguing because the HOMER1 protein links neural channels and receptors that the Aβ protein in the AD brain, and circHOMER1, was found to be significantly associated with AD diagnosis, clinical neurological staging, and dementia severity (Beylerli et al., 2024). circHOMER1 may have a direct role in AD regulating the expression of presenilins 1 (PSEN1) and 2 (PSEN2) by binding its predicted sites for mir-651 (Urdánoz-Casado et al., 2021). Undoubtedly, glia activation-mediated neuroinflammation is crucial for the advancement of AD and contributes to neuronal damage (Al-Ghraiybah et al., 2022; Uddin and Lim, 2022). Therefore, it has been suggested that several circRNAs may contribute to the inflammation and neuronal damage seen in AD patients or AD-like models. A small number of studies comparing CSF samples from AD patients to controls discovered a relationship between the disease's progression and risk factors and the circRNA expression profile (Shen X. et al., 2022). In short, top circRNAs interaction in AD development is demonstrated in Figure 3.

Moreover, circ-PCCA was found to be lower in the CSF of AD patients. This may be directly related to the pathophysiology of AD, as its overexpression helped to mitigate the severity of AD by blocking tau phosphorylation, an AD histopathological hallmark, and sponging mir-138-5p (Li Y. et al., 2020). circ-LPAR1 was found to be significantly expressed in AD patients by Wu et al.; this finding was also supported by the analysis conducted by Li et al. In this well-done study, they looked at the circLPAR1 regulatory axis, explaining how this circRNA might aid in Aβ-induced neuronal damage (Li Y. et al., 2020). The circLPAR1 sponged on mir-212-3p resulted in an upregulation of its target ZNF217 and accelerated the *in vitro* apoptosis, inflammation, and oxidative stress caused by Aβ25-35. Zinc finger protein 217 (ZNF217) expression increased in AD patients whereas mir-212-3p expression decreased (Wu et al., 2021). Using the same mouse model, circNF1-419 bound to Adaptor protein 2 B1 (AP2B1) and Dinamin-1, influencing several signaling pathways, especially at the synapse. This resulted in reduced expression of AD markers such as Tau, p-Tau, Aβ1-42, and APOE, which improved senile dementia (Diling et al., 2019). Even though much remains to be discovered regarding the precise regulatory function of circRNAs in autophagy processes and the underlying mechanisms, the authors proposed that circNF1-419 may be useful for dementia diagnosis and treatment (Diling et al., 2019). The circular RNA neurofibromin 1–419 (circNF1–419), which was produced from the neurofibromin 1 (NF1) gene, was bound by the dynamin-1 and adaptor protein 2 B1 (AP2B1) proteins in the cerebral cortex of neonatal rats (Diling et al., 2019). Through the use of the proteins Dynamin-1 and AP2B1, the PI3K-I/Akt-AMPK-mTOR, and PI3K-I/Akt-mTOR signaling pathways regulated autophagy activity (Diling et al., 2019). The list of top highlighted circRNA in the AD pathogenesis is listed in Table 3.

**Table 3:** Role of top studied circRNAs in the AD pathogenesis.

**CircRNAs in AD**	**Target**	**Function**	**References**
CDR1as	miR-7/ UBE2A	Regulating the amyloid peptide clearance	Memczak et al., 2013; D'Ambra et al., 2019; Shao et al., 2023
CircNF1-419	AP2B1	Affecting the AD marker protein levels through age-related signaling pathways.	Diling et al., 2019
CircHDAC9	miR-138 promote the expression of the target gene sirtuin 1 (Sirt1), which	Increased the content of Aβ and further led to synaptic and learning/memory deficit damage in neuronal	Lu et al., 2019
CircCwc27	Pur-α	Knockdown of circCwc27 reduced the expression level of APP and the production of Aβ *in vitro*	Song et al., 2022
CircHOMER1	miR-651, targeting presenilin-1 (PSEN1) and presenilin-2 (PSEN2) genes to	Affect the development of neuronal synapses, novel markers of AD risk, and diagnosis	Urdánoz-Casado et al., 2021
CircCORO1C	miR-105-binding APP and SNCA	Reduced the accumulation of Aβ and SNCA in neurons and alleviated the pathological process of AD. 4	Dube et al., 2019
circHDAC9	miR-142-5p	Alleviated Aβ42 formation	Lu et al., 2019
CircAβ-a	–	Cleaved into Aβ-peptides, play a role in the pathogenesis of sporadic AD	Mo et al., 2020
circ_0131235	IGF2R	AD biomarker	Bigarré et al., 2021
circTulp4	–	Potential AD biomarker for AD pathogenesis	Shen X. et al., 2022
mmu_circRNA_013636	–	Potential AD biomarker for AD pathogenesis	Huang et al., 2018b
mmu_circRNA_017963	–	Associated with different autophagosome and vesicular transport pathways	Huang et al., 2018a
circDLG1	–	AD plasma biomarker	Cochran et al., 2021
circDOCK1	–	AD plasma biomarker	Dube et al., 2019
circ_0000950	mir-103	Involved in neuroinflammation	Yang et al., 2019
circLPAR1	mir-212-3p / ZNF217	Sped up apoptosis, inflammation, and oxidative stress *in vitro*.	Wu et al., 2021
circ-PCCA	138-5p	Inhibiting Tau phosphorylation	Li Y. et al., 2020
mmu_circ_0000672	mmu-miR-351-5p cystatin F (Cst7)	Reduced the phagocytic ability of activated microglia to promote clearance of Aβ.	Ofengeim et al., 2017
mmu_circ_0001125	mmu-miR-351-5p cystatin F (Cst7)	Reduced the phagocytic ability of activated microglia to promote clearance of Aβ.	Zhang et al., 2021

## CircRNAs in amyotrophic lateral sclerosis

Amyotrophic lateral sclerosis (ALS) is a complex and devastating condition characterized by the progressive degeneration of motor neurons (Mead et al., 2023; Panchalingam and Kasivelu, 2024). The primary pathological characteristics of ALS, a heterogeneous neurodegenerative disease, are the degeneration of upper motor neurons that innervate neurons in the brainstem and spinal cord, as well as lower motor neurons that innervate muscles from the brainstem or spinal cord with no clear underlying mechanisms (Colantoni et al., 2023). The characteristics of TDP-43 proteinopathy were present in ALS patients, including the loss of TDP-43 in the nuclei of neuronal cells and cytoplasmic aggregates with dense or skeletal morphology in residual motor neurons (Jo et al., 2020). Recent studies have begun to uncover the role of circRNAs in the pathophysiology of ALS, suggesting that these molecules may hold key insights into disease progression and potential therapeutic avenues. For example, recent study indicating the connection of fused-in sarcoma (FUS) RNA-binding protein regulation including the FUSR521C and FUSP525L and circRNAs levels alternation in ALS. Indicating that the deregulation of circRNAs can be attributed to altered splicing dynamics due to mutated or absent FUS (Errichelli et al., 2017).

Therefore, circRNAs are thought to play a critical role in the disease's progression and pathology. Research has demonstrated that circRNAs are differentially expressed in ALS patient samples compared to healthy controls, indicating that they may contribute to neurodegeneration. For example, certain circRNAs have been shown to influence the expression of genes associated with neuronal survival and inflammation factors that are crucially involved in ALS pathology. Moreover, the dysregulation of specific circRNAs may reflect the underlying mechanisms of genetic and sporadic forms of ALS, providing insights into the disease's heterogeneity (Panchalingam and Kasivelu, 2024).

One notable circRNA is circSLC8A1, which has been identified as being significantly upregulated in ALS. Studies suggest that circSLC8A1 may interact with miRNAs known to be involved in neuronal function and stress responses (Hanan et al., 2020). By sequestering these miRNAs, circSLC8A1 could potentially alter their regulatory effects on mRNA targets that are crucial in maintaining motor neuron integrity. This mechanism highlights the potential for circRNAs to influence neuronal health and underscores their relevance in ALS research.

CircRNAs most likely have a role in the pathological control of ALS (Panchalingam and Kasivelu, 2024). Among the many dysregulated circRNAs found in ALS patients were 151 downregulated circRNAs, most of which were linked to the disease process of ALS (Colantoni et al., 2023; Panchalingam and Kasivelu, 2024). For example, CircHdgfrp3 contributes to the preservation of neuronal integrity and function in neuronal cells (D'Ambra et al., 2021). It could also result in abnormal accumulation of cytoplasmic FUS protein and increased mitochondrial translocation, which could cause excessive mitochondrial fission and damage (D'Ambra et al., 2021). Additionally, hsa_circ_0000567 derived from histone methyltransferase SETD3, controls mouse muscle differentiation (Eom et al., 2011). hsa_circ_0023919 deriving from the PICALM gene is also involved in clathrin-mediated endocytosis at neuromuscular junctions (Tebar et al., 1999). The relationship between the possible association of circRNAs with clinical data and the expression levels of circRNAs was also assessed (Dolinar et al., 2019). At the time of blood collection, there was a negative correlation found between the age of the patient and hsa_circ_0023919, hsa_circ_0000567, and hsa_circ_0088036 (Dolinar et al., 2019).

Compared to phosphorylated neurofilament heavy chain and neurofilament light chain, the most representative biomarkers in ALS, the sensitivity and specificity of these three circRNAs were as high as 90% in ALS patients (Dolinar et al., 2019). The hsa_circ_0088036, derived from the SUSD1 gene has been linked to ALS, Elevated levels of these circRNAs have been found in patients with ALS, for which hsa_circ_0088036 is already considered a biomarker (Dolinar et al., 2019). In conclusion, circRNAs represent a promising area of investigation in the field of neurodegenerative diseases, particularly in understanding the complexities of ALS.

### CircRNAs in Huntington's disease

The neurological condition known as Huntington's disease (HD) is inherited and causes symptoms that are emotional, cognitive, and physical (Ferguson et al., 2022). HD is a progressive neurodegenerative disorder caused by an expansion of CAG repeats in the HTT gene, leading to the production of an abnormal huntingtin protein. This disease is characterized by motor dysfunction, cognitive decline, and psychiatric symptoms, typically manifesting in mid-adulthood, however, the exact molecular bases of HD are not fully understood (Tabrizi et al., 2020).

Recent advances in molecular biology have shed light on several non-coding RNAs, particularly circRNAs, and their potential roles in various pathophysiological processes, including HD's pathology based on their unique properties (Gantley et al., 2023).

One proposed mechanism by which circRNAs may contribute to HD is through their interaction with the expanded CAG repeat region in the HTT gene. Studies have demonstrated that some circRNAs can bind to the mutant huntingtin protein, potentially influencing its aggregation and toxicity. The misfolding and aggregation of huntingtin are hallmarks of HD; thus, circRNAs that interfere with these interactions could represent a novel therapeutic target. Moreover, circRNAs are also involved in regulating the expression of genes linked to neuronal health. For instance, certain circRNAs have been identified as modulators of neurotrophic factors, which are crucial for the survival and function of neurons. Disruption of circRNA expression in HD may lead to decreased levels of neuroprotective signals, exacerbating neuronal vulnerability and contributing to the neurodegenerative process. By restoring normal circRNA function, it may be possible to enhance neuronal resilience and improve outcomes for individuals with Huntington's disease. For example, a recent study indicated that arginine-glutamic acid dipeptide repeats gene (RERE) is the source of the majority of the downregulated circRNAs in HD, and mutation of this gene can cause brain development (Ferguson et al., 2022), or the circHTT (2–6), a circRNA from the HTT gene that includes exons 2, 3, 4, 5, and 6, is more prevalent in the brains of HD patients than in healthy controls and has a strong positive correlation with CAG repeat length (Ayyildiz et al., 2023).

In another study, the overexpressing mutant HD murine model, 23 different circRNAs were dysregulated functioning in MAPK, dopaminergic synapses, and depression (Marfil-Marin et al., 2021).

Or the genome-wide investigations on mouse knock-in neural progenitors series clearly showed a more than 500 differently expressed circRNAs (Tabrizi et al., 2020; Marfil-Marin et al., 2021; Ayyildiz et al., 2023).

## CircRNAs in schizophrenia

Schizophrenia, a complex neuropsychiatric disorder marked by a range of cognitive, behavioral, and emotional dysfunctions, has long intrigued researchers seeking to unravel its intricate etiology, which includes wide range of symptoms, typically manifests in individuals between the ages of 16 and 30 (Mead et al., 2023). It is more prevalent in men than in women and has long been the focus of substantial research (Lobo et al., 2022). Recently, circular RNAs (circRNAs) have emerged as a promising area of study, based on their unique role, known for their stability and regulatory functions, influencing gene expression and neuronal activity. A recent genome-wide association study (GWAS) has found 287 genomic loci and at least 600 genes potentially related with schizophrenia, indicating that the majority of these genetic variants are in non-coding regulatory regions of the genome. Among the various molecular players implicated in this condition, circular RNAs (circRNAs) have emerged as significant contributors in neurodevelopment and synaptic plasticity, processes that are often disrupted in individuals with schizophrenia (Liao et al., 2022). Research has identified several circRNAs that exhibit altered expression levels in the brains of schizophrenia patients compared to healthy individuals. For instance, circRNA_104075 has been shown to be significantly upregulated in the prefrontal cortex of individuals with schizophrenia. This specific circRNA is believed to interact with microRNAs involved in neuronal signaling pathways, suggesting a potential mechanism through which altered circRNA expression could contribute to the neurological dysfunction observed in schizophrenia (Li Z. et al., 2020). Moreover, circRNAs may also influence the expression of genes linked to dopamine signaling, a neurotransmitter system widely implicated in the disorder's pathology. In addition to their role in gene regulation, circRNAs have been implicated in inflammatory responses within the central nervous system.

Neuroinflammation is increasingly recognized as a crucial component of schizophrenia, and circRNAs may modulate the inflammatory response by acting as molecular sponges for pro-inflammatory miRNAs. This suggests that targeting circRNAs could provide a novel therapeutic approach to mitigate neuroinflammation associated with schizophrenia, potentially alleviating some of the cognitive and emotional symptoms experienced by patients (Hatzimanolis et al., 2025). Based on the most data decrease in circRNA levels in patients' brains worldwide is usually related with schizophrenia, this decrease might make miRNAs more bioavailable, which would enhance their ability to inhibit target mRNAs and dysregulate protein expression. Patients with schizophrenia show different expression patterns for ADAR1 and QKI, two important RBPs involved in circRNA biogenesis, which may indicate that their production of circRNA is impaired (Huang et al., 2023; Hatzimanolis et al., 2025).

A case-control study conducted on peripheral blood for detection of circRNAs in schizophrenia has been nominated (hsa circRNA 103704, hsa circRNA 104597, and hsa circRNA 101836) with diagnostic significance in evaluation of schizophrenia, however since each patient had only been treated with standardized antipsychotic medications for a brief period of time further research is needed to prove the results and identifying other dysregulated circRNAs impacting on schizophrenia behavioral and mental diseases (Lobo et al., 2022). Despite the promising findings linking circRNAs to schizophrenia, much remains to be understood regarding their precise mechanisms of action and broader implications. Future research should aim to elucidate the network of interactions involving circRNAs, their corresponding target genes, and how these interactions are altered in the context of schizophrenia. Additionally, the development of circRNA-based biomarkers could facilitate early diagnosis and treatment monitoring, offering hope for more personalized therapeutic strategies (Sabaie et al., 2021).

### Potential clinical applications of circRNAs

Because of their abundance, stability, and specificity, circRNAs have the potential to be both novel therapeutic targets and diagnostic tools. Research on the abnormal expression of circRNAs and the ensuing dysregulation in their normal functioning in neurological conditions is gaining traction, and exciting new findings are being made (Vromman et al., 2023). Over the past 20 years, new knowledge about circRNA and how it interacts with regulatory mechanisms supporting complex and often highly polygenic neurological conditions like depression, schizophrenia, and bipolar disorder, as well as neurodegenerative diseases like Parkinson's and Alzheimer's disease, has emerged. The widespread expression of circRNAs in the brain is one of their most attractive features for researching neurological disorders. Where they exhibit other cell-type-specific expression patterns and high expression in neuronal cells relative to other cell types (He J. et al., 2019). CircRNAs can help create more effective RNA-based treatments because of their high stability, distinct transcript segments across the back splice junction region, and generally low immunogenicity (Wang et al., 2022). Plasma-derived circRNAs have also demonstrated promise for creating diagnostic biomarkers and panels devoted to the early detection and monitoring of neurological conditions, as circRNA detection has become more efficient (Liao et al., 2022). The presence of circRNAs in cerebrospinal fluid highlights their versatility as biomarkers in neurological conditions, providing a useful diagnostic window into the milieu of the central nervous system. Since certain circRNAs exhibit regulatory patterns that allow them to be translated into protein sequences a thorough comprehension of the mechanisms underlying their translational capacity may aid in the creation of RNA-based treatments that are more reliable and efficient. CircRNAs have several benefits over mRNA therapies, such as increased stability, longer translation times, and decreased immunogenicity (Liao et al., 2022; Guz et al., 2023).

Since certain circRNAs exhibit regulatory patterns that allow them to be translated into protein sequences, a thorough comprehension of the mechanisms underlying their translational capacity may aid in the creation of RNA-based treatments that are more reliable and efficient. CircRNAs have several benefits over mRNA therapies, such as increased stability, longer translation times, and decreased immunogenicity. CircRNA-based vaccines have recently been put forth as novel approaches to producing long-lasting and efficient expression of cancer and viral antigenic proteins. CircRNAs encoding the SARS-CoV-2 spike protein and circRNAs encapsulated in the charge-altering releasable transporter (CART) that encode antigens to target cancers are two examples (Qu et al., 2022).

### Challenges and future directions

Despite of the fact that the discovery of circRNAs and their multifaceted roles represents a paradigm shift in our understanding of RNA biology, especially regarding to their potential clinical usage in neurological disorders based on its high stability compared to mRNA, longer lifespan, their cell-specific expression profiles in neurodevelopment and normal brain function, and capacity to encode functional peptides, still several questions remain unanswered.

The precise mechanisms through which circRNAs exert their effects are still being elucidated, requiring future studies to explore the dynamic interactions between circRNAs, miRNAs, and mRNAs in the context of neuronal health and disease. Additionally, *in vivo* studies aimed at manipulating circRNA levels could provide critical insights into their roles in disease models and potentially open new avenues for therapeutic intervention (Kristensen et al., 2017). With machine learning techniques and larger sample sizes profiling different biofluids and cellular biopsies, including, patient-derived cells, and tissue cultures, they may be used as biomarkers for diagnostics. However, more research is needed to determine the exact mechanisms and effects of circRNA functions in brain homeostasis, as well as their contributions to the etiology of neuropsychiatric and neurodegenerative conditions (Kristensen et al., 2017). Furthermore, breaking through the blood-brain barrier and creating specific delivery methods and lack of standardized protocols for circRNAs detection are other remained difficulties for considering circRNAs as the effective tool for gene therapy of the brain in clinical applications, and filling these gaps need more developed diagnostics and treatments for customized treatments of neurological conditions (Gong et al., 2025).

## Conclusion

Highly abundant circRNAs in the brain, with their great potential as biomarkers due to their stability, diversity, specific expression patterns, and their key function in regulation of nervous system makes them excellent candidates for studying the neurological disorders specially AD. Unlike traditional therapies that often target symptoms rather than underlying mechanisms, circRNA-based strategies could address the molecular causes of the disease. Therefore, targeting specific circRNAs or their interactions with miRNAs might emerge as a therapeutic approach to mitigate neurodegeneration diseases via developing therapies that either bolster protective circRNA functions or inhibit those promoting AD pathology.

However, despite the promising outlook, several challenges regard their safety and efficacy must be rigorously evaluated in preclinical and clinical settings to ensure they provide real benefits to patients in clinic. Therefore, a deeper understanding of circRNA biology, their precise roles in AD, and the development of effective delivery systems will be crucial for translating research findings into clinical applications to significantly improve the quality of life for millions affected by AD.
